# Epigenetic Regulation of Ferroptosis in Inflammation: Molecular Mechanisms and Therapeutic Perspectives

**DOI:** 10.3390/ijms27188427

**Published:** 2026-09-21

**Authors:** Nana Li, Chonkit Lio, Zhenlian Lu, Jinfang Luo

**Affiliations:** 1 Department of Basic Medicine, Department of Pharmacy, Key Laboratory on the Property & Effect of Chinese Medicine (Ethnic Medicine), Guizhou Genuine Herbs Center of Consistency of Utility, Guizhou Key Laboratory of Miao Medicine, Guizhou University of Traditional Chinese Medicine, Guiyang 550025, China; linana@stu.gzy.edu.cn (N.L.); luzhenlian@gzy.edu.cn (Z.L.); 2 Faculty of Chinese Medicine, Macau University of Science and Technology, Avenida Wailong, Taipa, Macao SAR 999078, China; 3 State Key Laboratory of Quality Research in Chinese Medicine, Macau University of Science and Technology, Avenida Wailong, Taipa, Macao SAR 999078, China

**Keywords:** traditional Chinese medicine, epigenetics, ferroptosis, anti-inflammatory

## Abstract

Ferroptosis is an iron-dependent form of regulated cell death characterized by the lethal accumulation of lipid peroxides and has emerged as an important contributor to the initiation, progression, and amplification of inflammatory responses. DNA methylation, histone modifications, and noncoding RNA-mediated regulation influence ferroptosis by modulating multiple molecular pathways, thereby shaping the inflammatory microenvironment. Active constituents and compound formulas of traditional Chinese medicine (TCM) exhibit multi-target, multi-pathway regulatory properties that enable the simultaneous regulation of epigenetic modifications, ferroptosis, and inflammation. However, the molecular mechanisms linking these processes have not been systematically summarized. This review discusses the molecular mechanisms underlying ferroptosis, the epigenetic mechanisms that regulate ferroptosis, and the reciprocal interplay between ferroptosis and inflammation. It further highlights recent advances in understanding how active constituents and compound formulas of TCM modulate ferroptosis through epigenetic regulation to alleviate inflammatory injury. By integrating current evidence, this review provides mechanistic insights into the epigenetic basis of the anti-inflammatory actions of TCM and underscores the therapeutic potential of epigenetic modulation of ferroptosis for the treatment of inflammatory diseases.

## 1. Introduction

Inflammation is an essential host defense response that protects against pathogens, tissue injury, and other harmful stimuli. When this response is excessive, it can become a driver of disease. Chronic low-grade inflammation contributes to autoimmune, metabolic, cardiovascular, gastrointestinal, neurodegenerative, and malignant disorders [1,2,3]. Current anti-inflammatory treatments often lose efficacy during long-term use.

Ferroptosis is a regulated form of cell death defined by iron dependence and lipid peroxide accumulation [4]. Hallmarks include iron overload, depletion of glutathione (GSH), accumulation of lipid reactive oxygen species (ROS), and characteristic mitochondrial changes, such as shrinkage, increased membrane density, and reduced cristae [5,6]. Ferroptosis is closely connected to inflammation. Ferroptotic cells release damage-associated molecular patterns that activate innate immune pathways, whereas inflammatory cytokines can alter iron metabolism and antioxidant defenses, increasing cellular sensitivity to ferroptosis. These reciprocal effects may generate a feed-forward loop in chronically inflamed tissues.

Epigenetic regulation provides one route by which this loop is established. DNA methylation, histone modifications, and non-coding RNAs (ncRNAs) can alter the expression of ferroptosis-related genes without changing the DNA sequence. These mechanisms affect antioxidant defense, iron storage and transport, lipid metabolism, and inflammatory signaling [7,8]. Recent work suggests that aberrant epigenetic regulation can suppress genes such as glutathione peroxidase 4 (GPX4) and solute carrier family 7 member 11 (SLC7A11), alter iron homeostasis, and promote lipid peroxidation, thereby linking epigenetic dysregulation to ferroptosis-associated inflammation [9,10,11].

Traditional Chinese medicine contains multiple active constituents that can act on inflammatory and oxidative stress pathways. Increasing evidence indicates that some TCM-derived compounds and formulations also regulate ferroptosis, either directly or through epigenetic mechanisms [12,13]. This review focuses on these mechanisms. We first summarize the molecular links among epigenetics, ferroptosis, and inflammation. We then discuss studies in which TCM active constituents or compound formulas modulate ferroptosis-related inflammatory injury through DNA methylation, histone modification, or non-coding RNA pathways. Finally, we address current challenges and future perspectives in translating these findings into therapeutic strategies for inflammatory diseases.

**Figure 1:**
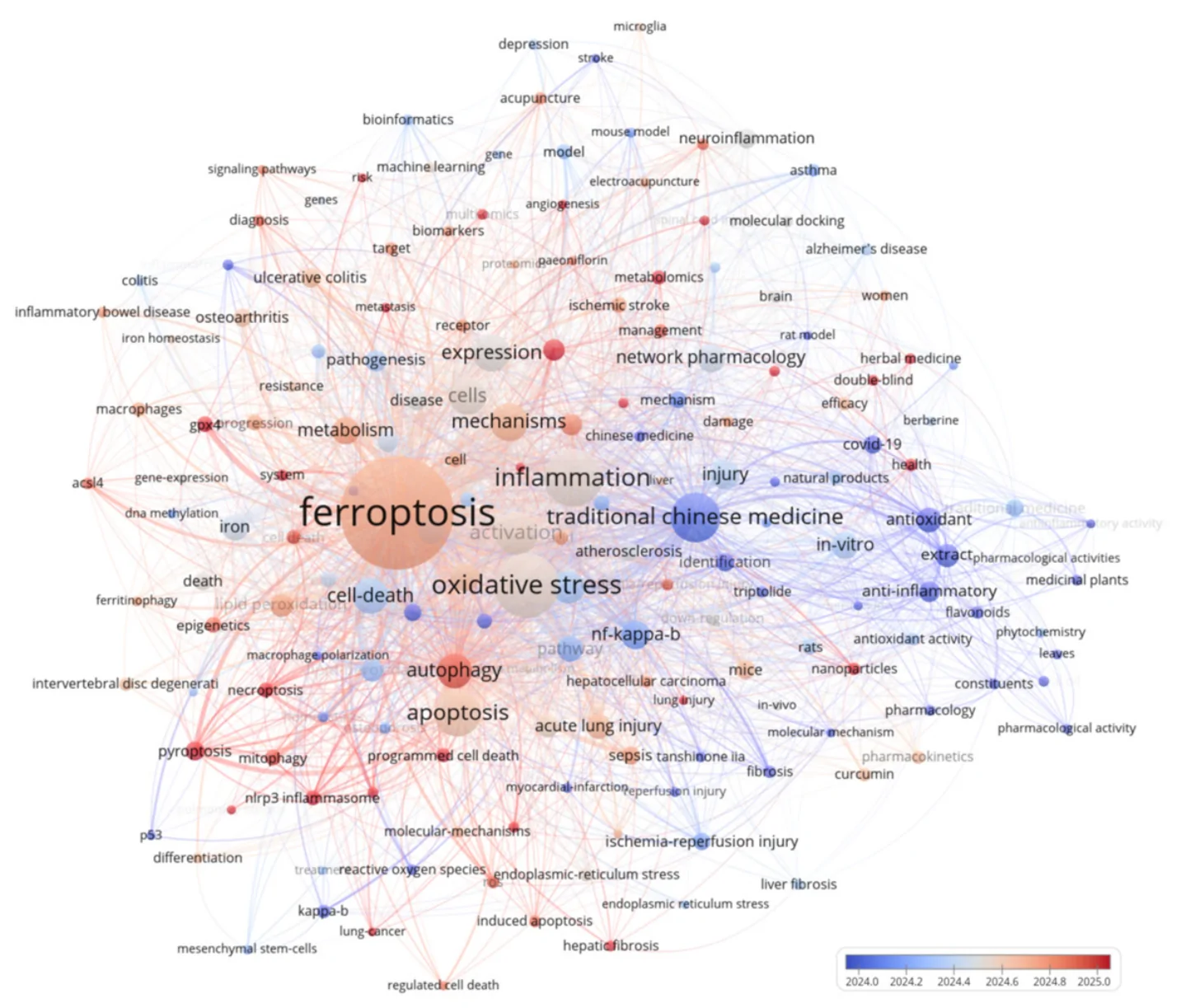
VOSviewer keyword co-occurrence network illustrating major research themes and their temporal evolution in studies investigating ferroptosis, inflammation, epigenetic regulation, and traditional Chinese medicine. Node size represents keyword frequency, line thickness indicates co-occurrence strength, and node color reflects the average publication year.

To provide an overview of the current research landscape, tab-delimited files were exported from the Web of Science using designated keywords, and a VOSviewer version 1.6.20-based keyword co-occurrence analysis was performed. The resulting network is presented in Figure 1, and the detailed data are provided in Table S1. The network is organized around ferroptosis, inflammation, oxidative stress, and TCM, suggesting that these themes form the main framework of the current review.

## 2. Molecular Associations Among Epigenetics, Ferroptosis, and Inflammation

### 2.1. Molecular Machinery Determining Ferroptosis Sensitivity

Ferroptosis sensitivity is governed by the antagonism between lipid peroxidation drivers and cellular antioxidant defenses. The principal antioxidant axis is the GPX4-dependent pathway, which reduces phospholipid hydroperoxides using glutathione as a cofactor [14]. Parallel GPX4-independent defenses include the ferroptosis suppressor protein 1 (FSP1)/coenzyme Q10 (CoQ10) axis [15], the dihydroorotate dehydrogenase (DHODH)/CoQ10 axis [16], and the GTP cyclohydrolase 1 (GCH1)/tetrahydrobiopterin (BH4) pathway [17]; inhibition of these antioxidant nodes markedly sensitizes cells to ferroptosis. Conversely, long-chain acyl-CoA synthetase 4 (ACSL4) esterifies polyunsaturated fatty acids (PUFAs) into PUFA-coenzyme A (CoA), thereby supplying substrates for lipid peroxidation [18]. Lysophosphatidylcholine acyltransferase 3 (LPCAT3) incorporates PUFA-CoA into membrane phospholipids, particularly phosphatidylethanolamine, generating the peroxidation-prone phospholipid species that drive ferroptotic membrane damage [18]. Sterol O-acyltransferase 1 (SOAT1) diverts PUFA-CoA toward cholesteryl esters, a second ACSL4-dependent route of PUFA utilization that can also fuel lipid peroxidation, although its net effect on ferroptosis sensitivity appears context-dependent [19]. By contrast, monounsaturated fatty acids are incorporated into membrane phospholipids in an ACSL3-dependent manner, displacing PUFAs and conferring ferroptosis resistance [20].

Iron availability and lipid droplet (LD) dynamics further modulate this sensitivity. Intracellular iron uptake is mediated by transferrin receptor (TFRC)-dependent endocytosis, followed by six-transmembrane epithelial antigen of the prostate 3 (STEAP3)-mediated Fe^3+^ reduction and divalent metal transporter 1 (DMT1/SLC11A2)-dependent release into the labile iron pool (LIP) [21]. Excess iron is sequestered by ferritin—composed of ferritin heavy chain 1 (FTH1) and ferritin light chain (FTL)—and exported by ferroportin (FPN/SLC40A1). Nuclear receptor coactivator 4 (NCOA4)-mediated ferritinophagy mobilizes stored iron to enhance ferroptosis sensitivity, a process restrained by E3 ubiquitin ligase HECT and RLD domain-containing E3 ubiquitin–protein ligase 2 (HERC2)-dependent NCOA4 degradation under iron-replete conditions. Dysregulated NCOA4 activation causes iron overload that drives Fenton chemistry and sensitizes cells to ferroptosis [22]. Finally, lipophagy releases LD-stored lipids to fuel peroxidation and increase sensitivity, whereas LD biogenesis sequesters neutral lipids and confers ferroptosis resistance.

### 2.2. DNA Methylation and Demethylation in Ferroptosis

DNA methylation is a major epigenetic mechanism controlling gene expression. It occurs mainly at CpG dinucleotides and is enriched in CpG islands within promoter regions. DNA methyltransferases (DNMTs) transfer methyl groups from S-adenosylmethionine (SAM) to generate 5-methylcytosine (5mC) [23,24]. Stable methylation patterns depend on DNA methyltransferase activity, demethylation pathways, histone-modifying enzymes, one-carbon metabolism, DNA integrity, and cell proliferation status. Disruption of any of these processes can change transcriptional output and cellular functions [25].

In inflammatory settings, promoter methylation can influence ferroptosis sensitivity. DNMT3A- and DNMT3B-mediated de novo methylation, followed by DNMT1-dependent maintenance, may repress ferroptosis-protective genes such as GPX4, SLC7A11, and FSP1, or their upstream transcriptional activator, nuclear factor erythroid 2-related factor 2 (Nrf2). Loss of these defenses favors iron-dependent lipid peroxidation and ferroptotic cell death [26].

Notably, DNA methylation itself carries no inherent pro- or anti-ferroptotic direction; its effect is determined entirely by the function of the methylated target. Methylation of ferroptosis-protective genes (GPX4, SLC7A11, FSP1) removes antioxidant defenses and sensitizes cells to ferroptosis, whereas methylation of pro-ferroptotic genes such as ACSL4 would be predicted to have the opposite effect. This principle is directly illustrated by the TCM literature summarized below: Asperosaponin VI, cordycepin, and berberine alleviate ferroptosis by reversing hypermethylation of protective loci, whereas ginsenoside Rg3 instead relieves ACSL4 promoter methylation to promote ferroptosis in hepatic stellate cells. Thus, although all of these constituents converge on the same epigenetic machinery, they drive ferroptosis in opposite directions depending on the target cell; both directions ultimately converge on the alleviation of disease progression. The same epigenetic machinery thus yields opposite therapeutic outcomes depending on whether the disease context demands ferroptosis inhibition or ferroptosis induction.

**Figure 2:**
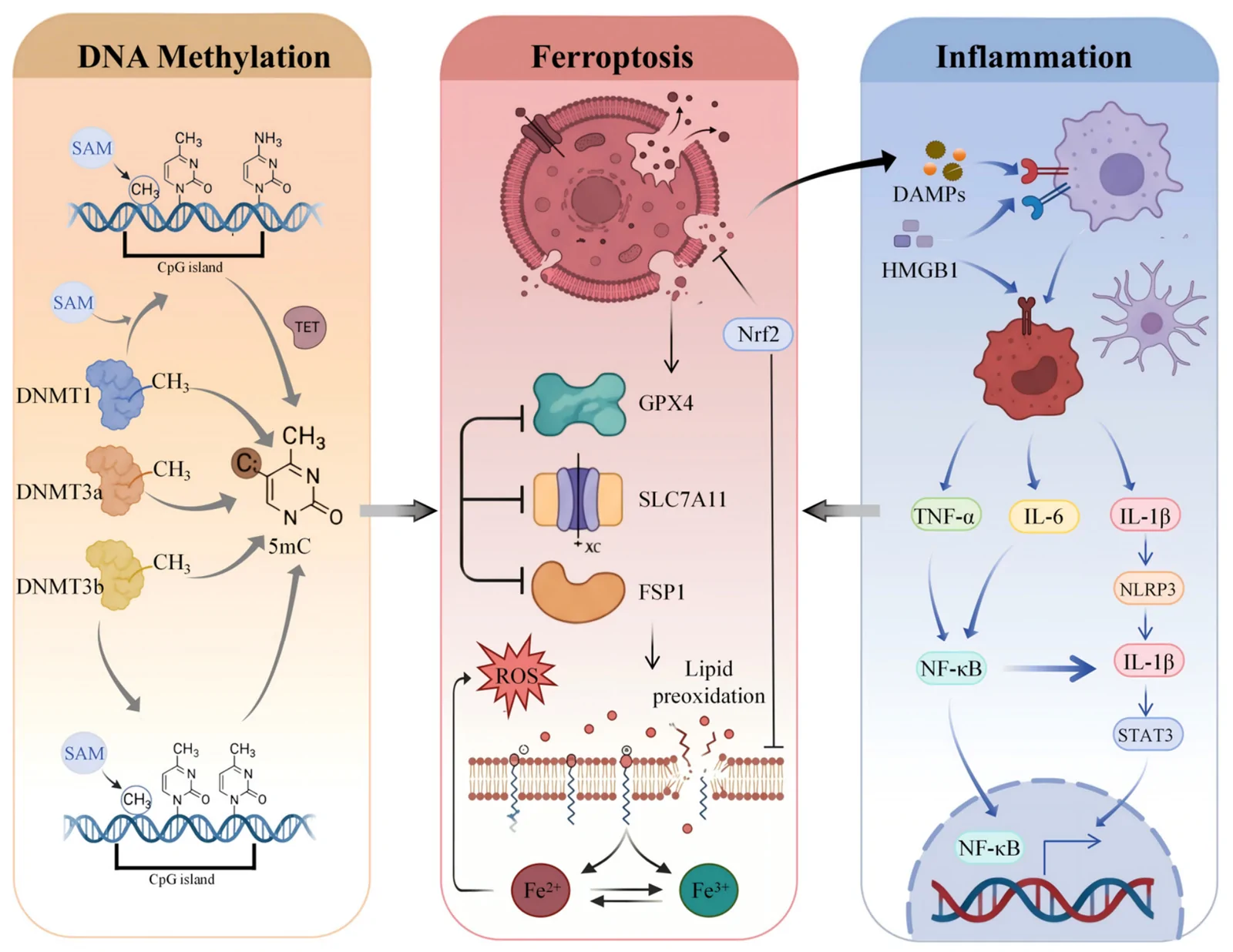
Mechanistic diagram illustrating the interplay among DNA methylation, ferroptosis, and inflammation.

This relationship is summarized in Figure 2, which shows how DNMT-mediated DNA methylation can suppress antioxidant defenses, promote lipid peroxidation, and connect ferroptotic cell death with inflammatory signaling.

### 2.3. Histone Modifications and Chromatin Regulation

Histone modifications regulate ferroptosis by changing chromatin accessibility at ferroptosis-related genes. Histone methylation provides several examples. EZH2-mediated H3K27me3 deposition at the NRF2 locus can repress NRF2 transcription and weaken antioxidant defense. G9a/GLP-mediated H3K9me2 modifications can downregulate SLC7A11 and GPX4 expression, whereas demethylases such as lysine-specific demethylase 3B (KDM3B), which removes H3K9me2, and lysine-specific demethylase 4A (KDM4A) activate SLC7A11 by removing H3K9me3 [27,28,29].

Histone acetylation also contributes to ferroptosis control. PCAF increases H3K9ac at the NRF2 locus and enhances NRF2 expression, whereas histone deacetylases (HDACs) reduce GPX4 transcription by deacetylating histones at the GPX4 promoter. Histone deacetylase inhibitors, including sodium butyrate, may also reduce NF-κB/NLRP3-associated inflammatory responses [30,31].

Other histone marks extend this regulatory network. H2Bub1 can activate SLC7A11 transcription, whereas the p53-ubiquitin-specific peptidase 7 (USP7) axis reduces H2Bub1 occupancy at SLC7A11 regulatory regions and promotes ferroptosis [32]. Histone lactylation has also been linked to ferroptosis and inflammation. In sepsis-associated lung injury, H3K18la regulates methyltransferase-like 3 (METTL3) and promotes ferroptosis through m6A modification of ACSL4 mRNA [33]. Lactate can also promote HMGB1 lactylation and acetylation in macrophages, increasing HMGB1 release and amplifying TLR4/NF-κB/NLRP3 signaling. Together, these findings suggest that histone modifications do not simply regulate ferroptosis genes; they also connect ferroptotic cell death to inflammatory amplification.

Across the histone-modification studies reviewed here, a consistent principle emerges: ferroptosis sensitivity reflects the balance of opposing enzymatic activities at specific loci rather than the presence of any single mark. Repressive H3K27me3 (EZH2) and H3K9me2 (G9a/GLP) at NRF2 or SLC7A11 lower antioxidant capacity, whereas activating H3K9ac (PCAF) and H2Bub1 at the same or related loci raise it; the net direction in a given cell depends on which writers/erasers dominate. This explains why HDAC inhibitors can be anti-inflammatory in some settings (restoring GPX4 acetylation and dampening NF-κB/NLRP3) yet potentially pro-ferroptotic in others. It also implies that therapeutic strategies targeting a single enzyme (e.g., EZH2 inhibition) must be evaluated against the possibility of compensatory shifts in the opposing activity.

**Figure 3:**
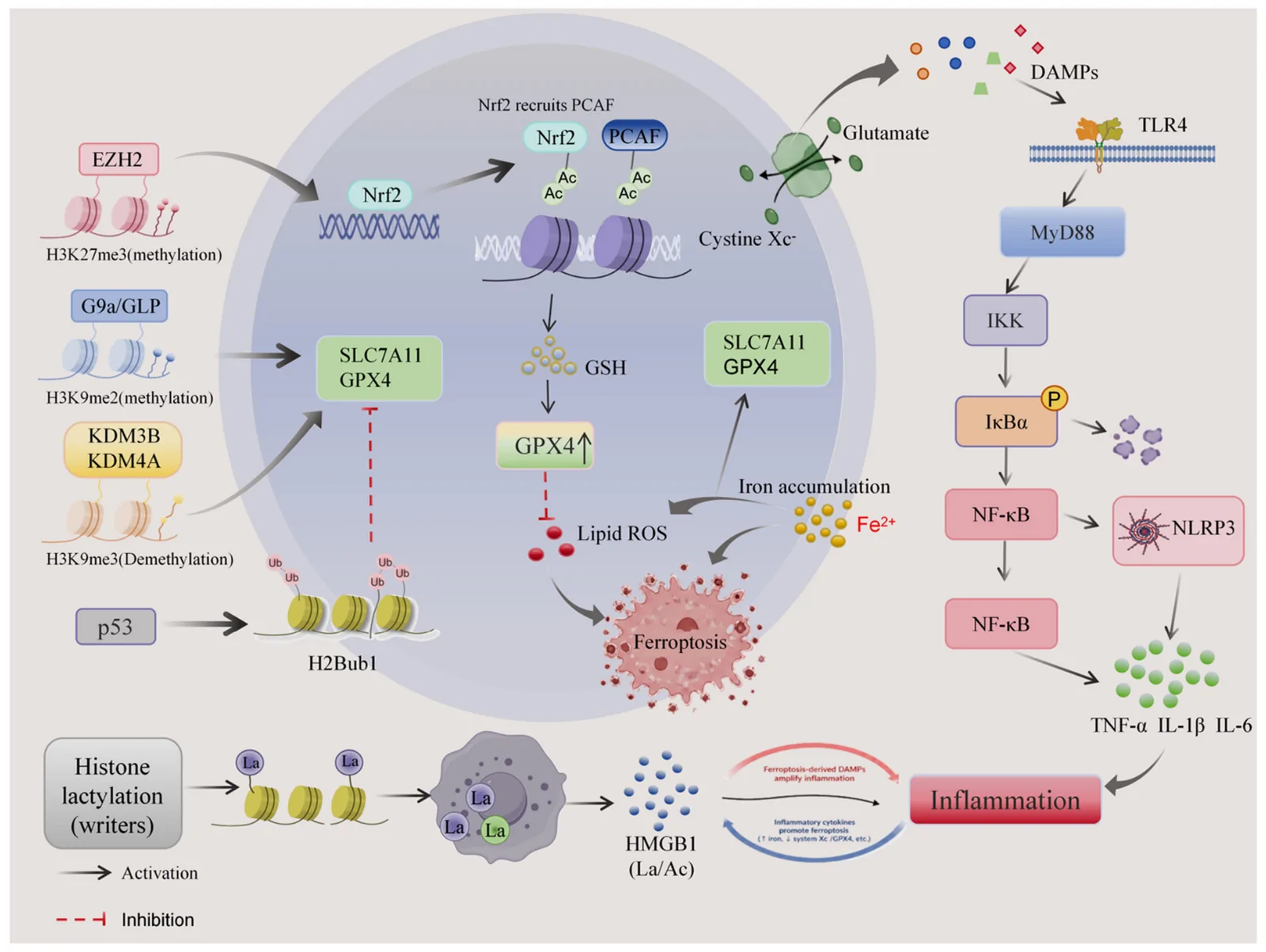
Mechanistic diagram illustrating the interplay among histone modifications, ferroptosis, and inflammation. Solid arrows indicate activation/promotion.

As summarized in Figure 3, histone methylation, acetylation, ubiquitination, and lactylation regulate ferroptosis-related genes and also influence DAMP release, NF-κB signaling, and inflammasome activation.

### 2.4. Non-Coding RNA-Mediated Regulation

ncRNAs are transcribed RNA molecules that do not encode proteins. They include microRNAs (miRNAs), long non-coding RNAs (lncRNAs), and circular RNAs (circRNAs) [34,35]. Many non-coding RNAs regulate ferroptosis. For example, miRNAs can bind the 3′-UTR of genes such as GPX4, SLC7A11, and ACSL4, leading to translational repression or mRNA degradation [36]. Another example is lncRNAs, which often act through competing endogenous RNA (ceRNA) mechanisms. For instance, LINC00618 has been linked to autophagy-dependent ferritin degradation and ferroptosis regulation [37], whereas NEAT1 has been reported to modulate ferroptosis through autophagy-related pathways [38]. CircRNAs can function as miRNA sponges; exosomal circ-ITCH from bone marrow stromal cells has been reported to enhance NRF2 activity and protect against high glucose-induced endothelial ferroptosis [39].

These RNA-based mechanisms also intersect with inflammatory signaling. Specific miRNAs and lncRNAs can modulate TLR4, NF-κB, or NLRP3 components, linking ncRNA networks to inflammation. Additionally, ferroptotic cells release HMGB1, ATP, and other damage-associated molecular patterns (DAMPs), which activate signaling cascades such as the TLR4/NF-κB pathway and trigger NLRP3 inflammasome assembly. The resulting cytokines, including TNF-α and IL-6, can further modulate SLC7A11 and GPX4 expression through NF-κB/STAT3-dependent mechanisms in a context-specific manner [40].

As with DNA methylation, ncRNAs have no fixed directional role in ferroptosis: miRNAs that target GPX4, SLC7A11, or FSP1 promote ferroptosis, whereas those targeting ACSL4 or TFRC suppress it. The ceRNA architecture (lncRNA/circRNA–miRNA–mRNA) adds a competitive, threshold-like property absent from enzyme-catalyzed modifications—small changes in sponge abundance can switch the output nonlinearly. A further layer is bidirectional crosstalk with inflammatory signaling: ferroptotic cells release HMGB1/ATP that activate TLR4/NF-κB, while inflammatory cytokines feed back onto SLC7A11/GPX4 expression via NF-κB/STAT3, potentially creating a self-reinforcing loop in chronically inflamed tissues. Whether this loop is initiated by epigenetic priming or by iron/lipid stress itself remains unresolved.

**Figure 4:**
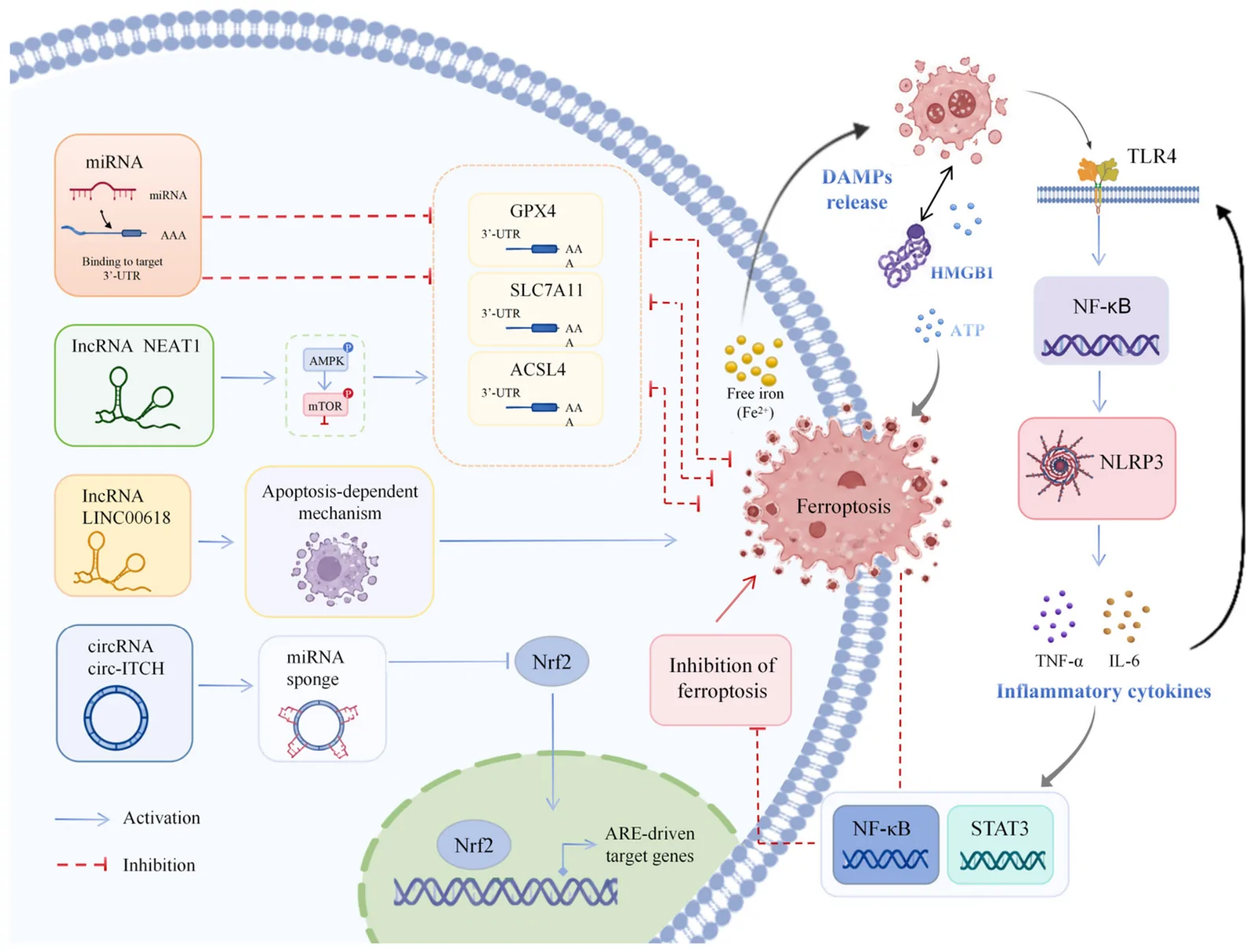
Mechanistic diagram illustrating the interplay among non-coding RNAs, ferroptosis, and inflammation. *“AAA” denotes the poly(A) tail of mRNA and “ARE” denotes the antioxidant response element*.

The regulatory network connecting non-coding RNAs, ferroptosis, and inflammation is summarized in Figure 4. miRNAs, lncRNAs, and circRNAs act through distinct mechanisms but converge on ferroptosis-related genes and inflammatory pathways.

## 3. Therapeutic Modulation of Epigenetically Regulated Ferroptosis

### 3.1. Pharmacological and Epigenetic Therapeutic Strategies

#### 3.1.1. Direct Modulation of Ferroptosis

Therapeutic modulation of ferroptosis centers on ferroptosis-inducing compounds (FINs), classified by molecular target. Class I FINs—including erastin, imidazole ketone erastin (IKE), sulfasalazine, and sorafenib—inhibit system Xc^−^, depleting cystine and GSH to indirectly inactivate GPX4 [41]. Class II FINs such as RSL3, ML162, and ML210 directly bind the selenocysteine active site of GPX4, causing lethal lipid hydroperoxide accumulation [42]. Class III FINs (e.g., FIN56) deplete mevalonate-derived CoQ10 and promote GPX4 degradation [6]. Beyond these three classes of FINs, the FSP1/CoQ10 axis has emerged as an additional druggable node that acts in parallel to GPX4 [15]: iFSP1 directly inhibits FSP1 enzymatic activity [43], whereas icFSP1 disables FSP1 by triggering its phase separation into inactive condensates [44]. Notably, unlike classical FINs, FSP1 inhibitors do not robustly induce ferroptosis on their own but rather sensitize cells to ferroptosis, particularly in the context of concomitant GPX4 inhibition [43].Conversely, iron chelators (deferoxamine, deferiprone) sequester labile Fe^2+^, while lipophilic antioxidants (ferrostatin-1, liproxstatin-1) scavenge lipid reactive oxygen species (ROS), each conferring ferroptosis resistance [41].

#### 3.1.2. Targeting DNA Methylation

DNMT inhibitors (DNMTis) reverse aberrant promoter hypermethylation of ferroptosis genes (e.g., GPX4, SLC7A11, NFE2L2), which can restore ferroptosis resistance in certain contexts and may confer adaptive tolerance to FINs [25,30]. The nucleoside analogues 5-azacytidine (5-AZA) and decitabine are incorporated into DNA, trap DNMTs, and induce passive demethylation; both are FDA-approved for myelodysplastic syndromes and acute myeloid leukemia [45,46]. Next-generation agents such as SGI-110 (guadecitabine)—a dinucleotide prodrug of decitabine—offer improved plasma stability [47]. GSK3685032, a first-in-class non-nucleoside reversible DNMT1-selective inhibitor, induces robust hypomethylation with superior tolerability in preclinical models. Beyond reactivating ferroptosis suppressors, DNMTis enhance interferon-γ signaling and can upregulate PD-L1 expression in certain tumor contexts, providing a rationale for combination with immune checkpoint inhibitors [48]. However, this regulation is context-dependent and may vary across cancer types.

#### 3.1.3. Targeting RNA Modifications and Non-Coding RNAs

The m6A machinery and ncRNAs represent druggable epitranscriptomic nodes. In certain contexts, METTL3-mediated m6A modification stabilizes SLC7A11 and GPX4 mRNAs via specific reader proteins, thereby promoting ferroptosis resistance [49]. The METTL3 inhibitor STM2457, a SAM-competitive antagonist, was shown to suppress AML growth and leukemic stem cell maintenance in preclinical models [50], and subsequent studies have linked METTL3 inhibition to enhanced ferroptosis sensitivity in tumor cells [49]. ncRNAs also modulate ferroptosis: miR-9, miR-375, and miR-378a-3p reduce SLC7A11 expression to sensitize cells to ferroptosis, whereas miR-15a-5p and miR-182-5p promote ferroptosis by targeting GPX4 [36]. lncRNAs such as LINC00336 and FERO regulate ferroptosis through competing endogenous RNA mechanisms. Therapeutic strategies include antagomirs, miRNA mimics, antisense oligonucleotides (ASOs), and CRISPR/dCas9-based epigenome editing to modulate these RNA networks.

The therapeutic sections above reveal a central dilemma: almost every node can be argued for or against depending on the disease context. In cancer, the objective is to induce ferroptosis (system Xc^−^ inhibition, GPX4 inhibition, METTL3 inhibition, and epigenetic repression of SLC7A11, e.g., via the EZH2/DOT1L/H3K79me2 axis), whereas in inflammatory and ischemic disorders, the objective is to suppress it (through DNMT1 inhibition to restore GPX4, ALKBH5 activation, and iron chelation). Systemic administration of epigenetic drugs therefore carries a theoretical risk of moving ferroptosis sensitivity in the ‘wrong’ direction in off-target tissues. Context-selective delivery, disease-stage stratification, and biomarker-guided patient selection appear essential for translation.

### 3.2. Traditional Chinese Medicine-Derived Constituents and Formulations

#### 3.2.1. Active Constituents of TCM

Active constituents are the chemical basis of many pharmacological effects of TCM. Their structural diversity allows them to influence oxidative stress, inflammation, and cell death pathways [51]. Recent studies suggest that several TCM-derived compounds regulate ferroptosis through epigenetic mechanisms, although the strength of evidence varies across models and disease contexts.

Several compounds act through DNA methylation. Asperosaponin VI (AVI), derived from *Dipsacus asper* Wall., has been reported to bind and inhibit DNMT1 and DNMT3A. This reverses hypermethylation of the GPX4 promoter, restores GPX4 expression, and attenuates osteoblast ferroptosis in diabetic osteoporosis [52,53]. Baicalein, from *Scutellaria baicalensis* Georgi, inhibits DNMT1 and regulates methylation of the SCARA5 promoter, thereby influencing hepatic stellate cell ferroptosis and inflammatory response [54,55]. Cordycepin inhibits DNMT1, reduces Kruppel-like factor 15 (KLF15) promoter methylation, increases KLF15 and FTH1 expression, and suppresses renal tubular epithelial cell ferroptosis in acute kidney injury [56,57]. Berberine suppresses DNMT1 and DNMT3A, reduces KLF4 promoter hypermethylation, and ameliorates diabetic nephropathy-associated oxidative stress and ferroptosis [58,59]. Icariside II and isoquercitrin have also been linked to DNMT1 downregulation and protection against high glucose- or metabolic stress-associated ferroptosis [60,61,62,63].

Other constituents act through histone or RNA modifications. Curcumin reduces the expression of lysine methyltransferases, including EZH2, MLL1, G9a, and SET7/9, and promotes apoptosis and ferroptosis in wild-type p53 HCT116 cells [64,65]. Ginsenoside Rg3 increases miR-6945-3p, suppresses DNMT3B, reduces ACSL4 promoter methylation, and promotes hepatic stellate cell ferroptosis, with anti-fibrotic effects [66,67,68]. Scoparone stabilizes the m6A reader YTH N6-methyladenosine RNA-binding protein 2 (YTHDF2) and promotes Beclin-1 (BECN1) translation, which facilitates BECN1-SLC7A11 complex formation and induces hepatic stellate cell ferroptosis [69,70]. Platycodin D (PD) suppresses methyltransferase-like 16 (METTL16)-dependent m6A modification of nuclear protein 1 (NUPR1) mRNA and promotes ferroptosis in prostate cancer cells [71,72,73]. Artesunate (Art) increases YTH N6-methyladenosine RNA binding protein C2 (YTHDC2)-recognized m6A modification of autophagy-related 5 (ATG5) mRNA, promoting ferritinophagy and ferroptosis in hepatocellular carcinoma [74,75]. Gastrodin (GAS) regulates the PI3K/Akt/ALKBH5/glutamate–cysteine ligase modifier subunit (GCLM) axis and inhibits ischemic stroke-associated ferroptosis [76,77]. Curdione promotes methyltransferase-like 14 (METTL14)- and YTHDF2-dependent degradation of SLC7A11 and solute carrier family 3 member 2 (SLC3A2) mRNAs, leading to GSH depletion and ferroptosis in colorectal cancer cells [78,79]. Shikonin (SHK) enhances NOP2/Sun RNA methyltransferase family member 2 (NSUN2)-mediated m5C methylation of TFRC mRNA, resulting in iron overload and ferroptosis in acute myeloid leukemia cells [80,81,82]. Evodiamine inhibits H3K18la at the hypoxia-inducible factor 1-alpha (HIF1A) promoter and reduces GPX4 expression, thereby linking histone lactylation, angiogenesis, and ferroptosis [83,84,85].

Representative TCM-derived active constituents that regulate ferroptosis through epigenetic mechanisms are summarized in Table 1. Most reported compounds act through DNA methylation or RNA modification pathways, whereas direct evidence involving histone modifications remains more limited.

#### 3.2.2. Compound Formulas of TCM

Compared with studies on single compounds, the number of studies on TCM compound formulas remains limited, and most available data are preclinical. Niujiao Dihuang Jiedu Decoction promotes NOP2/Sun RNA methyltransferase family member 5 (NSUN5)-mediated m5C modification of SLC7A11 mRNA, increases SLC7A11 protein expression, and attenuates ferroptosis in acute-on-chronic liver failure models [86]. Xingnaojing (XNJ) has been reported to upregulate GPX4 through ALKBH5-mediated m^6^A demethylation of SLC7A11 mRNA and to protect against ferroptosis in acute ischemic stroke [87]. Heixiaoyao San activates the SIRT1/p53/SLC7A11 pathway, increases SLC7A11 and GPX4, reduces p53 and ACSL4, and alleviates neuronal ferroptosis in an Alzheimer’s disease model [88]. Fuzheng Kang’ai Formula downregulates EZH2 and DOT1L, reduces H3K79me2 at the SLC7A11 promoter, and promotes ferroptosis through the SLC7A11/GPX4 axis in a tumor context [89]. These studies indicate that compound formulas may influence both epigenetic regulation and iron–lipid peroxide homeostasis. However, most data remain preclinical. Future work should define the active components, confirm direct molecular targets, and determine whether the observed epigenetic changes are causal rather than correlative.

Representative TCM compound formulas and their corresponding epigenetic mechanisms are summarized in Table 2. Compared with single compounds, evidence for multi-herb formulations remains relatively limited, but these studies suggest that compound formulas may regulate ferroptosis through coordinated effects on epigenetic enzymes and ferroptosis-related targets.

## 4. Conclusions

Epigenetic regulation of ferroptosis is an emerging mechanism through which TCM may exert anti-inflammatory effects. Current studies have reported associations between these TCM interventions and altered expression of key ferroptosis-related genes, including SLC7A11, GPX4, ACSL4, and FTH1, concurrent with changes in DNA methylation, histone modifications, or RNA-based regulation. These mechanisms provide a coherent framework for connecting TCM pharmacology with inflammatory control.

At the same time, the field is still at an early stage. Many studies rely on disease models in which ferroptosis, epigenetic regulation, and inflammation change in parallel, but direct causal links are not always established. More rigorous experiments are needed, including target validation, rescue approaches, dose–response studies, pharmacokinetic analysis, and well-controlled in vivo models. Integrating epigenetics, ferroptosis biology, and TCM pharmacology should help identify which interventions hold translational potential and which mechanisms are most relevant to inflammatory diseases.

## Figures and Tables

**Table 1:** TCM active constituents regulating ferroptosis through epigenetic mechanisms. “↑” denotes upregulation/increase and “↓” denotes downregulation/decrease of the indicated molecule or modification.

TCM Constituent	Source	Regulatory Mechanism	References
Asperosaponin VI	*Dipsacus asper* Wall.	Inhibits DNMT1 and DNMT3A, reverses hypermethylation within the GPX4 promoter region, restores GPX4 expression, reduces inflammatory factor release	[52,53]
Baicalein	*Scutellaria baicalensis* Georgi	Inhibits DNMT1, reduces CpG island methylation within the SCARA5 promoter region, alleviates inflammation	[54,55]
Cordycepin	*Cordyceps militaris*	Directly inhibits DNMT1, reduces CpG island methylation within KLF15 promoter region, KLF15↑, promotes FTH1 expression, inhibits renal tubular epithelial cell ferroptosis	[56,57]
Berberine	*Coptis chinensis* Franch., *Phellodendron amurense* Rupr.	Inhibits DNMT1 and DNMT3A, prevents aberrant hypermethylation within KLF4 promoter region, KLF4↑, suppresses oxidative stress and ferroptosis marker	[58,59]
Icariside II	*Epimedium*	DNMT1↓, inhibits high glucose-induced podocyte ferroptosis, alleviates inflammatory responses and ECM accumulation	[60,61]
Isoquercitrin	*Bidens pilosa* L., *Morus alba* L.	DNMT1↓, GPX4↑, inhibits ferroptosis, improves insulin sensitivity	[62,63]
Curcumin	*Curcuma longa* L.	Downregulates lysine methyltransferase expression, reduces histone lysine methylation, triggers apoptosis and ferroptosis in a p53-dependent manner	[64,65]
Ginsenoside Rg3	*Panax ginseng* C. A. Mey.	Upregulates miR-6945-3p to inhibit DNMT3B, relieves methylation within the ACSL4 promoter region, promotes ACSL4 expression, induces HSC ferroptosis, exerts anti-inflammatory and anti-fibrotic effects	[66,67,68]
Scoparone	*Artemisia capillaris*, *Artemisia scoparia*	Binds to the Asn462 residue of YTHDF2 to enhance its stability, promotes BECN1 mRNA translation, forms the BECN1-SLC7A11 complex to suppress SLC7A11, induces HSC ferroptosis, attenuates liver fibrosis	[69,70]
Platycodin D	*Platycodon grandiflorum*	METTL16↓, reduces m6A modification of NUPR1 mRNA, inhibits NUPR1 expression, induces prostate cancer cell ferroptosis	[71,72,73]
Artesunate	*Artemisia annua* L.	Enhances Wilms tumor 1-associated protein (WTAP)-mediated m6A modification of ATG5 mRNA, promotes YTHDC2-dependent translation increases ATG5 protein synthesis, triggers ferritinophagy, induces ferroptosis	[74,75]
Gastrodin	*Gastrodia elata* Rchb. f.	Binds to PI3K, activating the PI3K/Akt pathway, phosphorylates Akt to induce ALKBH5 nuclear translocation, reduces m6A methylation of GCLM mRNA, restores GCLM expression, enhances GSH synthesis, inhibits ferroptosis	[76,77]
Curdione	Curcumae Rhizoma	METTL14 and YTHDF2↑, promotes m6A modification of SLC7A11 and SLC3A2 mRNA and their degradation by YTHDF2, reduces protein levels, leads to GSH depletion and decreased GPX4 activity, induces ferroptosis	[78,79]
Shikonin	*Lithospermum erythrorhizon* Sieb. et Zucc.	NSUN2↑, promotes m5C methylation of TFRC mRNA, enhances TFRC expression, leads to iron overload and lipid peroxidation, induces ferroptosis	[80,81,82]
Evodiamine	*Evodia rutaecarpa* (Juss.) Benth.	Inhibits H3K18la modification within the HIF1A promoter region, HIF1A↓, relieves transcriptional suppression of Sema3A; reduces GPX4 expression to induce ferroptosis, synergistically inhibits angiogenesis and immune evasion	[83,84,85]

**Table 2:** TCM compound formulas regulating ferroptosis through epigenetic mechanisms. “↑” denotes upregulation/increase and “↓” denotes downregulation/decrease of the indicated molecule or modification.

Drug Name	Composition	Regulatory Mechanism	References
Niujiao Dihuang Jiedu Decoction (NDD)	*Gentiana macrophylla* Pall., *Siphonostegia chinensis* Benth., *Artemisia capillaris* Thunb., *Gardenia jasminoides* J. Ellis, *Glycyrrhiza uralensis* Fisch.	Promotes NSUN5-mediated m5C methylation modification of SLC7A11 mRNA, SLC7A11↑, inhibits ferroptosis, alleviates hepatic histopathological injury and inflammatory state	[86]
Xingnaojing	Moschus, *Curcuma aromatica* Salisb., Borneolum, *Gardenia jasminoides* J. Ellis	Upregulates GPX4 expression through the demethylase ALKBH5/SLC7A11 pathway, alleviates inflammatory responses, protects against ferroptosis in acute ischemic stroke	[87]
Heixiaoyao San	*Rehmannia glutinosa*, *Bupleurum chinense* DC., *Angelica sinensis* (Oliv.) Diels, *Paeonia lactiflora* Pall., *Atractylodes macrocephala* Koidz., *Poria cocos* (Schw.) Wolf., *Zingiber officinale* Rosc., *Glycyrrhiza uralensis* Fisch., *Mentha haplocalyx*.	Activates the SIRT1/p53/SLC7A11 signaling pathway, SIRT1, SLC7A11, GPX4↑, p53, ACSL4↓, inhibits neuronal ferroptosis, ameliorates cognitive dysfunction in AD model rats	[88]
Fuzheng Kang’ai Formula	*Pseudostellaria heterophylla* (Miq.) Pax, *Atractylodes macrocephala* Koidz., *Astragalus membranaceus* (Fisch.) Bunge, *Hedyotis diffusa* Willd., *Solanum nigrum* L., *Salvia chinensis* Benth., *Iphigenia indica* (L.) A. Gray, *Coix lacryma-jobi* L., *Akebia trifoliata* (Thunb.) Koidz., *Rubus parvifolius* L., *Curcuma zedoaria* (Christm.) Roscoe, *Glycyrrhiza uralensis* Fisch.	FZKA Formula induces ferroptosis by epigenetically downregulating SLC7A11/GPX4 via the EZH2/DOT1L/H3K79me2 axis, thereby remodeling the tumor immune microenvironment and attenuating tumor-associated inflammation	[89]

## Data Availability

The keyword co-occurrence data (summarized in Table S1) were derived from publications retrieved from the Web of Science Core Collection using specified search terms. The exported tab-delimited files were processed with VOSviewer version 1.6.20 to generate the network analysis. Table S1, which contains the full co-occurrence matrix and network metrics, is provided as Supplementary Materials.

## Supplementary Materials

The following supporting information can be downloaded at https://www.mdpi.com/article/10.3390/ijms27188427/s1.
